# Dysfunction of the Neurovascular Unit in Ischemic Stroke: Highlights on microRNAs and Exosomes as Potential Biomarkers and Therapy

**DOI:** 10.3390/ijms22115621

**Published:** 2021-05-25

**Authors:** Timea Forró, Zoltán Bajkó, Adrian Bălașa, Rodica Bălașa

**Affiliations:** 1 2nd Clinic of Neurology, Târgu Mureș County Emergency Clinical Hospital, 540136 Târgu Mureș, Romania; 2 Doctoral School, ‘George Emil Palade’ University of Medicine, Pharmacy, Science, and Technology of Târgu Mureș, 540142 Târgu Mureș, Romania; 3 1st Clinic of Neurology, Târgu Mureș County Emergency Clinical Hospital, 540136 Târgu Mureș, Romania; bzoltan2003@yahoo.com (Z.B.); rodica.balasa@umfst.ro (R.B.); 4 Department of Neurology, ‘George Emil Palade’ University of Medicine, Pharmacy, Science, and Technology of Târgu Mureș, 540142 Târgu Mureș, Romania; 5 Clinic of Neurosurgery, Târgu Mureș County Emergency Clinical Hospital, 540136 Târgu Mureș, Romania; adrian.balasa@yahoo.fr; 6 Department of Neurosurgery, ‘George Emil Palade’ University of Medicine, Pharmacy, Science, and Technology of Târgu Mureș, 540142 Târgu Mureș, Romania

**Keywords:** neurovascular unit, exosomes, extracellular vesicles, microRNAs, stroke, brain ischemia, biomarkers, mesenchymal stem cells

## Abstract

Ischemic stroke is a damaging cerebral vascular disease associated with high disability and mortality rates worldwide. In spite of the continuous development of new diagnostic and prognostic methods, early detection and outcome prediction are often very difficult. The neurovascular unit (NVU) is a complex multicellular entity linking the interactions between neurons, glial cells, and brain vessels. Novel research has revealed that exosome-mediated transfer of microRNAs plays an important role in cell-to-cell communication and, thus, is integral in the multicellular crosstalk within the NVU. After a stroke, NVU homeostasis is altered, which induces the release of several potential biomarkers into the blood vessels. The addition of biological data representing all constituents of the NVU to clinical and neuroradiological findings can significantly advance stroke evaluation and prognosis. In this review, we present the current literature regarding the possible beneficial roles of exosomes derived from the components of the NVU and multipotent mesenchymal stem cells in preclinical studies of ischemic stroke. We also discuss the most relevant clinical trials on the diagnostic and prognostic roles of exosomes in stroke patients.

## 1. Introduction

Stroke is a leading cause of death and disability worldwide [1]. It is classically defined as a neurological deficit due to an acute focal injury of the central nervous system (CNS) by a vascular cause [2]. Ischemic stroke, the most common type of stroke, is caused by a narrowing or blockage in an artery that supplies the brain, which leads to interruption of blood flow in the corresponding territory [3], thereby causes irreversible cell damage in the ischemic core through oxygen deprivation and increased inflammation [4]. The peri-infarct zone, also called the penumbra, contains cells at risk of further damage, but can potentially be rescued if appropriate interventions are available [5]. Although plentiful neuroprotective agents have been studied in the past decades [6], timely reperfusion, including intravenous thrombolysis with tissue plasminogen activator (alteplase) [7] and thrombus removal by mechanical thrombectomy [8] remains the only immediate treatment option in acute ischemic stroke management.

The prognosis of ischemic stroke is highly dependent on the interaction between clinical and demographic factors, such as age or stroke severity [9]. The addition of biological information, such as blood biomarkers or genetic polymorphisms, could further enhance the prognostic value of clinical data. A reliable outcome prediction method might improve decision-making processes and may be useful to improve and personalize stroke care [10].

Emerging data suggest that exosomes, which are nanosized (30–150 nm) endosome-derived vesicles, can be useful diagnostic, therapeutic, and prognostic markers in stroke [11]. Along with shedding microvesicles or ectosomes (10–1000 nm) and apoptotic bodies (50–5000 nm), exosomes represent one of the three main subtypes of the extracellular vesicles (EVs) [12]. The biogenesis of exosomes is a finely calibrated process that includes four stages: initiation, endocytosis, multivesicular body formation, and exosome secretion, which is the process in which these multivesicular bodies fuse with the plasma membrane [13]. After being released from their cells of origin, exosomes can interact by endocytosis, fusion, or ligand–receptor interactions with recipient cells [14].

Secreted by most human cells, exosomes contribute to intercellular signaling via cell-to-cell communication in both physiological and pathological processes, and function in different CNS diseases, such as stroke [15,16]. They also have the ability to cross the blood–brain barrier (BBB), thereby helping in the exchange between the CNS and the peripheral circulation [17]. Their lipid bilayer and aqueous core allow for the transport of various molecules, including proteins, lipids, and nucleic acids from their parent cells to adjacent or distant recipient cells through paracrine or autocrine mechanisms [18]. The exosomal contents are published and continuously updated by multiple databases, including Vesiclepedia [19], EVpedia [20,21], and Exocarta [22,23]. Proteins that are uniquely found in exosomes, such as tetraspanins (CD9, CD63, CD81, CD82), heat-shock proteins (Hsc70, Hsp90), proteins involved in membrane transport and fusion (GTPases, annexins, flotillin), in MVB biogenesis (Alix, TSG101), or related to lipids and phospholipases are often considered to be markers for exosomes [24]. Exosomes are abundantly present in most body fluids, including blood (serum or plasma), cerebrospinal fluid, urine, breast milk, saliva, amnion fluid, semen, nasal secretion, bronchoalveolar lavage, synovial fluid, bile, aqueous humor, and also in biological fluids produced under various disease conditions [25,26].

Valadi et al. first suggested that exosome-mediated transfer of microRNAs (miRs) could be a new mechanism of cell-to-cell communication [27]. Compared with cellular and free miRs, these exosomal forms have several particular characteristics that define them in CNS homeostasis and diseases. They are more accessible, have distinct expression patterns, are more stable against degradation, and act as nanocarriers to deliver miRs and siRNAs to the CNS. Moreover, the brain’s cellular and tissue status can be directly monitored by CNS-derived exosomal miRs through information carried from injured neural parent cells [14].

An increasing number of studies have emphasized the key role of the neurovascular unit (NVU) in ischemic stroke [28]. The NVU is a functional and morphological multicellular entity involving neurons, glial cells (oligodendrocytes, astrocytes, microglia), extracellular matrix, and brain vessels (pericytes, endothelial cells, vascular smooth muscle cells) [29], which acts to maintain homeostasis within the brain microenvironment [30]. After an ischemic insult, the resulting hypoxia not only induces anaerobic metabolism, but also contributes to the release of pro-inflammatory cytokines from glia and neurons leading to neuroprotection or further neurotoxicity in the brain [31]. Glial cells are the primary components of the peri-infarct area with important roles in post-stroke immune regulation. A large number of astrocytes, the most common glia type, survive after stroke and have regulatory effects in response to ischemia [32]. Furthermore, the NVU induces a coordinated response in order to preserve and re-establish blood flow and thereby diminish neuronal damage [30]. Activation of the NVU elicits the release of plentiful potential biomarkers into blood vessels, cerebrospinal fluid, and the extracellular space [33]. Moreover, through multiple interactions between the various components, the NVU has integrated function in neurovascular repair, inflammatory immune response, BBB regulation, and cell preservation during and after stroke [34]. Exosome secretion has been reported from all components of the NVU [35]; under ischemic conditions, specific types of exosomes exert neuroprotective effects [36].

The NVU concept appears to be a promising pathway in stroke patient care as a source of biomarkers representing all elements of this unit, but also as a potential constituent of new diagnostic protocols. Our review highlights the possible beneficial roles of exosomes released by the individual components of the NVU and their therapeutic potential when they are derived from mesenchymal stem cells (MSCs) in preclinical studies of ischemic stroke. We also present the most relevant publications of recent years on the diagnostic and prognostic roles of exosomes in clinical trials involving stroke patients.

## 2. Preclinical Studies of Exosomes Derived from NVU Components

The NVU concept emphasizes the importance of not only the neurons but also the glial and vascular components. Exosomes are essential in the communication between these elements. Brenna et al. characterized the different brain-derived EVs under physiological and ischemic conditions in mice. They found that microglia are the main contributors to the physiological pool of brain EVs, while after experimental stroke, an increase in astrocytic EVs was observed [37]. In the following sections, we detail the roles of exosomes in ischemic stroke from the NVU point of view, emphasizing every source of cells constituting this unit (see also Table 1).

### 2.1. Neural-Derived Exosomes (Ne-Exos)

Cortical neurons are able to release exosomes from their somato-dendritic compartments [38,39] in order to modulate local synaptic plasticity, trans-synaptic communication, post-stroke remodeling, and regeneration [35].

Song et al. reported the contribution of miR-181c-3p derived from cortical Ne-Exos to regulate neuroinflammation in rats after middle cerebral artery occlusion (MCAO). This effect was attributed to chemokine CXC motif ligand 1 (CXCL1), a target gene of miR-181-3p, which was downregulated by this miR in astrocytes [40]. Furthermore, miR-98 derived from Ne-Exos acts as a post-ischemic endogenous protective factor by inhibiting microglial phagocytosis via the targeting of platelet-activating factor receptor (PAFR), thereby attenuating ischemia-induced neuronal death [41]. In addition, Xu et al. revealed that neurons transfer miR-132 to endothelial cells via secreting exosomes. This miR maintains brain vascular integrity and regulates the expression of vascular endothelial cadherin (VE-cadherin) by targeting eukaryotic elongation factor 2 kinase (eef2k) [42].

Neural stem cells (NSCs) provide neurotrophic support in various CNS diseases [43]. Following ischemia, intravenous administration of NSC-derived exosomes (NSC-Exos) provides neuroprotection possibly by preserving astrocytic function [44], and improves functional recovery by reducing neuronal apoptosis and microglial density [45]. Moreover, NSC-Exos promote tissue, cellular, and functional outcomes including lesion volume decrease, hemispheric swelling, and brain atrophy in a thromboembolic mouse model of stroke [46]. Similar results were reported in a porcine ischemic stroke model by the same research group [47].

### 2.2. Exosomes Derived from Glial Components

#### 2.2.1. Oligodendrocyte-Derived Exosomes (Od-Exos)

Oligodendrocytes produce the myelin sheath and thus facilitate impulse conduction. Frühbeis et al. revealed a glutamate-dependent exosome release from oligodendrocytes mediated by Ca^2+^ through oligodendroglial N-methyl-D-aspartate (NMDA) and α-amino-3-hydroxy-5-methyl-4-isoxazolepropionic acid (AMPA) receptors. These vesicles transport different cargos to neurons, thereby participating in bidirectional glial–neuron communication and contributing to axonal integrity. Od-Exos also provide protection and metabolic support for neurons [48,49].

In addition, Fröhlich et al. reported that Od-Exos promote neuronal survival under conditions of oxygen–glucose deprivation (OGD), an in vitro model of cerebral ischemia. These exosomes appear to directly transfer antioxidant enzymes, such as catalase and superoxide dismutase 1 (SOD1), to neurons in order to enhance their stress tolerance. Moreover, they activate pro-survival signaling pathways, such as Akt and extracellular signal-regulated protein kinases 1 and 2 (ERK1 and ERK2) [50].

#### 2.2.2. Astrocyte-Derived Exosomes (As-Exos)

Astrocytes are the most numerous glial cell type of the CNS with crucial roles in the regulation of innate and adaptive immune responses [51]. Beyond their involvement in proinflammatory responses, they participate in BBB maintenance and permeability, synaptic regulation, neural survival, and growth support, with many of their functions being mediated by exosomes [35,52]. The aquaporin-4 (AQP4) water channels, which are highly expressed on the end-feet of astrocytes, play a key role in brain water homeostasis. Following ischemia, the increased expression of proinflammatory mediators and the resulting neurovascular inflammation lead to alterations in AQP4 expression causing cerebral edema [53]. Kitchen et al. revealed that targeting AQP4 not only reduces CNS edema but also leads to accelerated functional recovery following hypoxia [54]. Recently, Sylvian et al. confirmed that targeting these water channels is efficacious in reducing brain swelling during the early acute phase of cerebral ischemia using a photothrombotic stroke model in mice. A part of this effect was attributed to an extra-osmotic change in brain energy metabolism, as indicated by the increased levels of glycogen [55].

As-Exos contain numerous miR species that are substantially different from the miRs detectable in astrocytes, likely because of the existence of a mechanism that distributes certain miRs to exosomes [56]. Moreover, different extracellular stimuli can modify the cargo of As-Exos. In response to trophic (adenosine triphosphate, ATP) or anti-inflammatory (interleukin-10, IL-10) stimuli, As-Exos contain proteins involved in promoting neural survival, regulation of dendritic branching, neurite outgrowth, and synaptic transmission, while in response to an inflammatory stimulus (IL-1β), they involve proteins that participate in the regulation of peripheral immune response and immune cell trading to the CNS [57]. As-Exos subjected to oxidative and heat stress are also altered in their composition; they are specifically enriched in protective HSP70 [58].

Pei et al. reported that As-Exos ameliorate ischemia-induced damage and inhibit apoptosis through the suppression of autophagy in neurons subjected to OGD [59]. A follow-up study revealed the possible involvement of exosome-mediated miR-190b in this protection via the targeting of autophagy-related gene 7 (Atg7) [60]. Although excessive or insufficient levels of autophagy promote cell death, moderate levels are pro-survival in response to stressful circumstances [61]. Chen et al. demonstrated that exosome-shuttled circSHOC2, a circular RNA from ischemic pre-conditioned astrocytes, reduces neuronal apoptosis by targeting the miR-7670-3p/sirtuin 1 (SIRT1) axis to promote autophagy [62]. Other As-Exo-derived miRs with similar effects are miR-361 and miR-34c. Both of these miRs suppress cell apoptosis and alleviate nerve damage in rats with ischemia/reperfusion (I/R) injury: miR-361 targets cathepsin B (CTSB) and downregulates the AMP-activated protein kinase/mammalian target of rapamycin (AMPK/mTOR) signaling pathway [63], while miR-34c targets Toll-like receptor 7 (TLR7) to downregulate the nuclear factor-kappa B (NF-κB)/mitogen-activated protein kinase (MAPK) axis [64].

Hira et al. affirmed that inhibition of semaphorin 3A (Sema3A) in the subacute phase of stroke suppressed astrocyte activation and negatively regulated miR-30c-2-3p and miR-326-5p in As-Exos to promote axonal outgrowth and functional recovery in MCAO rats by increasing prostaglandin D_2_ synthase [65]. In addition, Wang et al. identified an oligomannose-binding protein, synapsin 1, which is released from astrocytes via exosomes under stress conditions such as ischemia, and which can modulate neuronal outgrowth and survival as well as neuron–glia interactions [66]. It was also suggested that intra-arterial administration of miR-133b-enriched MSC-derived exosomes enhances post-stroke neurological recovery, neurite outgrowth, and plasticity through a secondary release of As-Exos, possibly by downregulating the expression of Rab9 effector protein with Kelch motifs (RABEPK) in rats subjected to MCAO [67]. Under hypoxic and ischemic conditions, astrocytes are capable of releasing exosomes containing non-pathogenic prion proteins, thereby improving neuronal survival [68].

Brain ischemic preconditioning (IPC), the adaptation of the brain to lethal ischemia when first exposed to mild doses of a sub-toxic stressor, is protective against ischemic cerebral injury [69]. One possible mechanism of IPC for neuroprotection is associated with an exosome-mediated miR-92b-3p transport from ischemic preconditioned astrocytes to neurons, which attenuates OGD-induced neuronal apoptosis and injury [70].

#### 2.2.3. Microglia-Derived Exosomes (Mi-Exos)

Microglia are considered the principal immune cells of the brain; following ischemic injury, they are the first to respond to the damage [71]. Microglial activation is essential for synaptic remodeling, neurogenesis, and angiogenesis, thereby acting to improve functional recovery after stroke. It can be categorized into two different types: M1 or inflammatory phenotype, and M2 or anti-inflammatory phenotype. Depending on the activation phenotype, microglia can exert either cytotoxic or cytoprotective effects [72]. Mi-Exos modulate and spread inflammation, carry growth factors, and regulate synaptic activity [35].

Song et al. demonstrated that miR-124 derived from M2 Mi-Exos exerts neuroprotective effects by promoting neural survival and attenuating ischemic brain injury, neural deficits, and apoptosis via the targeting of ubiquitin-specific protease 14 (USP14). At 72 h after transient MCAO, this exosomal miR has been shown to be upregulated in the ischemic penumbra region [73]. The same research group reported that M2 Mi-Exos also reduce glial scar formation and improve post-stroke recovery via miR-124, which inhibits the migration and proliferation of astrocytes by reducing the expression of signal transducer and activator of transcription 3 (STAT3), a direct target of miR-124, and phosphorylated-STAT3, the activated form of STAT3. Moreover, this exosomal miR has been suggested to be involved in the induction of astrocyte-to-neural progenitor cell transition by increasing Sox2 and decreasing Notch1 expression [74].

Tian et al. investigated the protective role and pro-angiogenic effects of microglia in ischemic stroke. They found that IL-4-polarized microglia might increase endothelial cell tube formation by the secretion of exosomes that contain miR-26a [75]. In contrast, OGD-activated Mi-Exos induce significant brain microvascular endothelial cell (BMEC) damage and permeability through the upregulation of exosomal miR-424-5p by modulating the basic fibroblast growth factor (FGF2)/STAT3 pathway [76].

In a recent study, Raffaele et al. stated that the infusion of pro-regenerative Mi-Exos in the peri-infarct area restores protective microglia/macrophage functions, preventing their senescence at later stages of stroke, and enhances the maturation of G protein-coupled receptor 17 (GPR17)-expressing oligodendrocyte precursor cells (OPCs), thereby increasing functional recovery. Moreover, these exosomes exert their beneficial effects on the maturation of OPCs possibly via the transmembrane tumor necrosis factor-α/tumor necrosis factor receptor 2 (tmTNF/TNFR2) axis [77].

### 2.3. Exosomes Derived from Vascular Components

#### 2.3.1. Endothelial Cell-Derived Exosomes (Ec-Exos)

BMECs are a main cellular component of the BBB that form the interface between circulating blood and nervous tissue and regulate CNS homeostasis [78]. Exosomes derived from BMECs present receptors for macromolecule transport across the BBB and modulate immune and inflammatory responses [35]. They are particularly enriched with ribosomal proteins, histones, and proteins involved in exosome biogenesis and cell adhesion [79].

During acute ischemic brain injuries, vascular Ec-Exos are essential to neural repair and brain protection. Zhou et al. affirmed that intracerebral injection of Ec-Exos reduces infarct volume, improves neurological outcome, and promotes neurogenesis by activating neural progenitor cell proliferation and migration in the peri-infarct area, ipsilateral ventral sub-regions of subventricular zone, and dentate gyrus of the hippocampus in MCAO rats [80]. Another recent study demonstrated that brain Ec-Exos improve functional motor recovery and are pivotal for the regulation of synaptic plasticity and function. Moreover, they play a role in altering brain plasticity via miR-126-3p, which increases neurite outgrowth and protects PC12 cells from nerve damage and apoptosis [81]. Zhang et al. reported that ischemic and non-ischemic cerebral Ec-Exos promote axonal growth of cortical neurons by modulating miR-19a, miR-27a, miR-195, and miR-298 and targeting axon-inhibitory proteins such as Sema6A, phosphatase and tensin homolog (PTEN), and ras homolog family member A (RhoA) in recipient neurons. Moreover, ischemic Ec-Exos have a more potent effect in mediating axonal homeostasis and plasticity [82].

Endothelial progenitor cells (EPCs) are known to modulate the functions of endothelial cells [83]. Exosomes derived from these cells (EPC-Exos) protect endothelial cells against ischemia by improving angiogenic function and decreasing apoptosis and reactive oxygen species production. These protective effects are enhanced by miR-210 mainly through the promotion of mitochondrial function [84].

The beneficial roles of exosomes have been investigated in diabetic mice. Ec-Exo treatment promoted vascular, myelin, and axonal density in the ischemic boundary zone, as well as M2 macrophage polarization, thereby improving neurological and cognitive functional outcomes possibly mediated by miR-126 [85]. Enrichment with the same miR in EPC-Exos reduced acute injury by decreasing infarct volume and preserving cerebral microvascular density and blood flow, and improved neurological functional recovery by accelerating neurogenesis and angiogenesis through the downregulation of cleaved caspase-3 and upregulation of vascular endothelial growth factor receptor 2 (VEGFR2) [86]. Moreover, these miR-126-enriched exosomes modulate the protective effects of moderate exercise on the brain against ischemia in both acute and chronic stages of ischemic injury [87].

It was also reported that Ec-Exos from femoral arteries could directly protect neurons against I/R injury by suppressing ischemia-induced cell cycle arrest and apoptosis in SH-SY5Y nerve cells [88].

#### 2.3.2. Pericyte-Derived Exosomes (Pc-Exos)

Pericytes are isolated cells in close functional and anatomical contact with endothelial cells [89]. They exert trophic and neuroprotective activity and promote brain recovery, angiogenesis, and neurogenesis through exosome secretion [35]. These cells are also capable of generating MSCs in the perivascular area of lesioned or inflamed vessels [90]. Although hypoxic Pc-Exos have been shown to promote angiogenesis [91], to our best knowledge there is no relevant information in the literature regarding the possible roles of these exosomes during ischemic conditions.

## 3. Preclinical Studies of Mesenchymal Stem Cell-Derived Exosomes (MSC-Exos)

Restorative cell-based therapies, including intravenous administration of MSCs, improve functional outcomes after stroke [92]. The use of MSC-Exos as an alternative to MSCs offers several advantages, such as higher safety profile, less tumorigenicity, minimized occlusion of the microvascular system, lower immunogenicity, and the ability to cross biological barriers, i.e., the BBB [93]. They are mainly extracted from MSC subtypes, including bone marrow mesenchymal stem cells (BMSCs) and adipose-derived stem cells (ADSCs) [94].

Multiple studies suggested that MSC-Exos could be beneficial to promote post-stroke recovery due to their ability to mediate restorative effects involved in stroke, such as angiogenesis, neurogenesis, white matter restoration, oligodendrogenesis, and axonal sprouting (see also Table 2) [95].

### 3.1. Bone Marrow Mesenchymal Stem Cell-Derived Exosomes (BMSC-Exos)

Xin et al. demonstrated that intravenous administration of multipotent MSC-Exos improves functional recovery and neurovascular plasticity in rats subjected to MCAO. This effect was attributed to an exosomal delivery of miR-133b from BMSCs to neurons and astrocytes, which downregulates the expression of connective tissue growth factor (CTGF) and RhoA, thins the glial scar, and promotes neurite outgrowth [96,97,98]. Similarly, miR-17-92 cluster-enriched MSC-Exos increase functional recovery and neural plasticity after stroke possibly by targeting PTEN to activate the phosphatidylinositol-3-kinase (PI3K)/Akt/mTOR/glycogen synthase kinase 3 beta (GSK-3β) signaling pathway [99]. Additionally, these MSC-Exos enhance the axonal growth of cortical neurons by activating the same PTEN/mTOR signaling pathway [100].

Zhao et al. examined the anti-inflammatory effects of BMSC-Exos in acute brain ischemia. These exosomes suppressed cysteinyl leukotriene receptor 2 (CysLT_2_R)-ERK1/2-mediated M1 microglial polarization, promoted microglial conversion into M2 phenotype, increased secretion of anti-inflammatory molecules, and decreased production of pro-inflammatory cytokines, thereby markedly attenuating ischemic brain injury in MCAO rats [101]. The same research group reported that miR-233-3p derived from these exosomes promotes neurological deficits by improving learning and memorizing abilities, and attenuates cerebral ischemic injury through the inhibition of pro-inflammatory responses regulated by M1 microglial polarization via the targeting of CysLT_2_R [102]. Recently, Liu et al. reported that BMSC-Exos ameliorate cerebral ischemic injury by suppressing NLR family pyrin domain containing 3 (NLRP3) inflammasome-mediated inflammation and pyroptosis via the modulation of microglial polarization [103]. Furthermore, exosomal miR-138-5p derived from BMSCs reduces post-ischemic neurological impairment by inhibiting inflammatory responses and promoting the proliferation of astrocytes through the negative regulation of lipocalin 2 (LPCN2) [104]. In addition, miR-134-enriched BMSC-Exos suppress oligodendrocyte apoptosis by downregulating caspase-8 after OGD treatment [105]. Pan et al. revealed that the combination of miR-132-3p and MSC-Exos had beneficial effects on ameliorating ischemic brain injury. MiR-132-3p-enriched MSC-Exos protected endothelial cells from ischemia-induced apoptosis, oxidative stress, and tight junction disruption through the repression of protein p120 Ras GTPase-activating protein (RASA1) expression and activation of the Ras/PI3K/Akt/endothelial nitric oxide synthase (eNOS) signaling pathway [106]. Another recent study analyzed whether preconditioning of lithium-induced MSCs modifies exosome secretion patterns. Due to lithium treatment, MSC-Exos displayed increased levels of miR-1906, which inhibited the TLR4 and familiar proinflammatory signaling cascades, thereby enhancing neuroregeneration, neuroprotection, and neurological recovery in ischemic mice [107].

Doeppner et al. studied the therapeutic effects of BMSC-Exos compared with BMSCs after cerebral ischemia in mice, demonstrating that exosomes are not inferior to MSCs. They promote neuroregeneration and recovery, modulate peripheral immune responses, and induce long-term neuroprotection [108]. Moreover, Moon et al. revealed that treatment with BMSC-Exos was superior to that with BMSCs themselves. These exosomes contained various miRs essential for neurogenesis and angiogenesis, such as miR-184 and miR-210 [109]. It was also reported that administration of BMSC-Exos promoted recovery of fine motor function of the hand in monkeys after cortical injury, with a return to pre-injury levels within the first 3–5 weeks of recovery [110]. Moreover, Go et al. suggested that EVs derived from MSCs reduce neuroinflammation and enhance functional recovery after cortical injury in aged Rhesus monkeys by shifting microglia towards restorative functions [111].

Safakheil et al. examined the effect of BMSC-Exos in combination with rosuvastatin in MCAO rats. This combination therapy reduced cell death and neuroinflammation and promoted neuroprotection, thereby enhancing functional recovery after stroke [112].

### 3.2. Adipose-Derived Stem Cell-Derived Exosomes (ADSC-Exos)

It has been reported that miR-30d-5p and miR-126 levels are decreased in both patients and animal models of ischemic stroke [113,114]. ADSC-Exos enriched with miR-30d-5p prevent cerebral ischemic injury by suppressing autophagy and promoting M2 microglial/macrophage polarization [113]. Moreover, miR-126-overexpressing exosomes enhance neurogenesis and angiogenesis by increasing doublecortin and Von Willebrand factor levels and suppress microglial activation and inflammatory response induced by ischemia, thereby improving functional recovery [114].

Exosomal miR-181-5p derived from ADSCs promotes the angiogenesis of BMECs via the targeting of transient receptor potential melastatin 7 (TRPM7) after OGD in rats. Furthermore, these exosomes upregulate the expression of hypoxia-inducible factor 1α (HIF-1α) and VEGF and downregulate the expression of tissue inhibitor of metalloproteinase 3 (TIMP3) [115]. In addition, ADSCs inhibit apoptosis and inflammation by miR-21-3p suppression, which contributes to methionine adenosyltransferase 2B (MAT2B) upregulation, possibly mediated by exosomes [116]. Moreover, pigment epithelium-derived factor (PEDF)-overexpressing ADSCs-Exos also suppress apoptosis and activate autophagy to ameliorate cerebral ischemia [117]. Human ADSC-Exos increase the survival and proliferation of immortalized HT-22 hippocampal neuronal cells after brain injury [118].

Recently, Zhang et al. reported that miR-22-3p derived from ADSC-Exos attenuates apoptosis and brain ischemia by inhibiting the lysine demethylase 6B (KDM6B)-mediated bone morphogenic protein 2 (BMP2)/Bcl-2 modifying factor (BMF) axis in rats after I/R injury [119].

## 4. Exosomes in Stroke Patients

Multiple studies have investigated the diagnostic and prognostic role of exosomes and their cargos in acute ischemic stroke (AIS) patients [11]. One of the most common exosomal contents are miRs, and each of them have a distinct role in the various molecular pathways involved in stroke.

**Figure 1:**
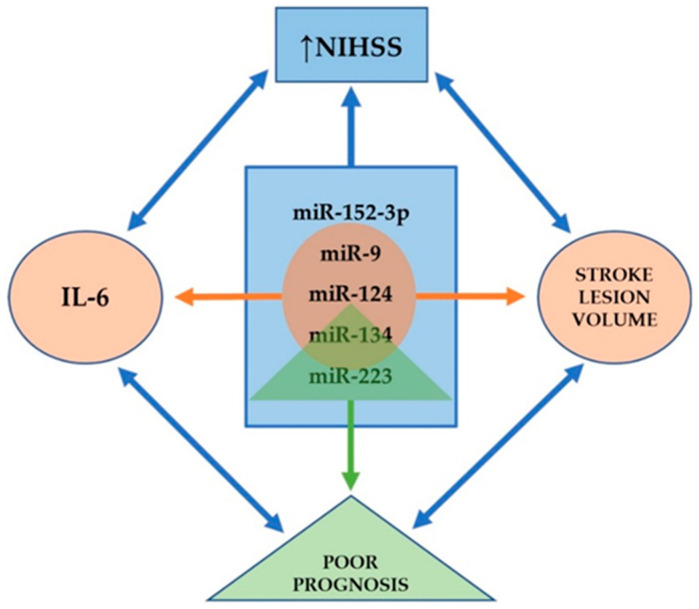
Exosomes as possible biomarkers in human studies. All the miRs listed in the central blue rectangle correlate to the NIHSS score. The three miRs listed in the orange circle correlate to infarct volume and serum concentrations of IL-6. The miRs in the green triangle are associated with poor prognosis. Nevertheless, all the elements representing clinical and paraclinical findings found around the blue rectangle correlate with each other.

Ji et al. found that serum levels of exosomal miR-9 and miR-124 are elevated in AIS patients: both of these miRs were correlated to National Institutes of Health Stroke Scale (NIHSS) scores, but also to infarct volume and serum concentrations of interleukin-6 (IL-6) [120]. These miRs are highly expressed in the CNS and enhance neurogenesis [121,122]; furthermore, they are promising biomarkers to evaluate the degree of damage caused by ischemic injury [120]. Another exosomal miR that is upregulated in AIS patients and correlates with stroke severity is miR-223. Chen et al. revealed that miR-223 was also associated with stroke occurrence and poorer short-term prognosis [123]. MiR-134 is a brain-specific, neuroprotective miR that plays an important role in dendritic spine growth control [124], neuritogenesis [125], and in memory and plasticity [126]. Zhoue et al. reported a significant increase in exosomal levels of miR-134 in AIS patients within 24 h of stroke onset: these levels were associated with infarct volume, NIHSS scores, and worse post-stroke prognosis, and additionally with the expression of serum IL-6 and plasma high-sensitivity C reactive protein (hs-CRP) [127]. In contrast, serum levels of exosomal miR-152-3p were significantly lower in AIS patients, especially in those with NIHSS scores ≥7. Furthermore, the lowest levels of miR-152-3p were found in cases of large-artery atherosclerosis, and these levels were significantly lower in the acute phase than in the chronic phase of stroke [128]. Taken together, miR-9, miR-124, miR-134, miR-152-3p, and miR-223 are associated with the severity of stroke; miR-134 and miR-223 with poor prognosis; miR-9, miR-124, and miR-134 with the infarct volume and the level of IL-6 (see Figure 1).

Two additional miRs that can be found in the exosomes of stroke patients are miR-21-5p and miR-30-5p. These are apoptosis-related miRs that can distinguish the hyperacute phase of ischemic stroke from the subacute and recovery phases [129]. Moreover, plasma exosomal miR-422a and miR-125b-2-3p were also demonstrated as monitoring and diagnostic markers of stroke patients, with the assertion that the combined use of these two may be powerful for determining stroke stage [130]. Progression of asymptomatic carotid artery stenosis with >50% luminal narrowing is considered a potential risk factor for stroke or transient ischemic attack during follow-up. Dolz et al. reported significantly higher expression of exosomal miR-199b-3p, miR-27b-3p, miR-130a-3p, miR-221-3p, and miR-24-3p in these patients [131].

Clinical assessments conducted on cell-free therapeutics remain extremely limited; however, there are plentiful in vitro and animal experiments exploring these therapies. MiR-124-overexpressing MSCs are among the rare exosome-based stroke curatives that have moved to phase I/II clinical trials (NCT03384433) [132].

Besides miRs, it has been demonstrated that exosomes released into the circulation after stroke can contain pro-inflammatory proteins, including CRP [133].

## 5. Future Directions and Conclusions

The NVU is a conceptual model involving neurons, glial cells, and brain vessels, having each of them a distinct contribution to the overall function of this structure. Exosomes are a newly defined way of interaction between these components due to their involvement in intercellular communication. Numerous studies discuss the role of these EVs secreted by the constituents of the NVU, however, these are almost exclusively involving animal research and are not supported by clinical evaluation.

Ischemic stroke is a major cause of morbidity and mortality worldwide. Despite advances in understanding the underlying pathophysiology, reperfusion is the only immediate treatment option for AIS patients. This justifies the unmet clinical need for further studies aimed to develop new therapeutics in this field. Recent advances in experimental approaches of drug discovery, including high-throughput screening [134] and computer-aided drug design [135], can provide novel insights into the identification and validation of potential therapeutic targets not only in various neurodegenerative diseases, but also in stroke. Moreover, these techniques can also serve as a basis for the target validation of exosomes in future studies.

Apart from the above-mentioned, in vitro models that can precisely simulate the NVU can help us to expand our understanding of the complex interactions between all components of this unit during ischemic stroke. Microfluidic organ-on-a-chip models, such as perfused BBB on-a-chip [136] and human brain microvessel-on-a-chip [137], have been demonstrated to be amenable for optical advanced imaging, which makes them useful in studying molecular transport mechanisms involved in the transcytosis of nanoparticles, viruses, or biologicals across the BBB. These platforms enable real-time monitoring of permeability changes, thereby offering an opportunity to examine the exosome’s release and penetration during stroke, as well as ischemia-induced neuroinflammation. Furthermore, 3D cultures and organoids are also capable to mimic BBB dysfunction thus might be potentially used in stroke modeling and therapy development [138]. There is recent preliminary preclinical evidence that cerebral organoid transplantation might be an effective intervention for stroke treatment [139].

Early stroke diagnosis and prognosis prediction are often challenging. Brain ischemia is a heterogeneous process that cannot be characterized by a single biomarker. Therefore, diagnostic panels should be composed of multiple biomarkers representing distinct pathophysiological processes, including inflammatory response, BBB disintegration and brain edema, necrotic and apoptotic cell death, oxidative stress, and thrombosis [33].

A better understanding of the pathological aspects of exosomes and their cargos will contribute to stroke diagnosis, outcome prediction, and therapy, and thus to the improvement of patient care. In the future, complex diagnostic stroke protocols should include not only clinical and neuroradiological findings, but also biomarkers representative of all elements of the NVU.

## Figures and Tables

**Table 1:** Prognostic and diagnostic markers carried within NVU component-derived exosomes.

NVU Component	Exosome Cargo	Target	Effects of Dysregulation	References
Neural-derived exosomes	miR-181c-3p	Downregulating CXCL1 in astrocytes	Protective effects on neuroinflammation	Song et al. [40]
	miR-98	Targeting PAFR	Inhibit microglial phagocytosis; Ameliorate ischemia-induced neuronal death	Yang et al. [41]
	miR-132	Regulating VE-cadherin by targeting eef2k	Maintain vascular integrity	Xu et al. [42]
Oligodendrocyte-derived exosomes	Antioxidant enzymes (catalase and SOD1)	Activating pro-survival pathways (Akt, ERK1, and ERK2)	Promote neuronal survival; Enhance stress tolerance	Fröhlich et al. [50]
Astrocyte-derived exosomes	miR-190b	Targeting Atg7	Inhibit apoptosis; Suppress autophagy; Ameliorate ischemia-induced neuronal damage	Pei et al. [59,60]
	circSHOC2	Acting on the miR-7670-3p/SIRT1 axis; Regulating autophagy	Inhibit apoptosis; Promote autophagy; Ameliorate neuronal damage	Chen et al. [62]
	miR-361	Downregulating the AMPK/mTOR pathway and targeting CTSB	Suppress cell apoptosis; Ameliorate nerve damage	Bu et al. [63]
	miR-34c	Downregulating the NF-κB/MAPK axis by targeting TLR7	Suppress cell apoptosis; Ameliorate nerve damage	Wu et al. [64]
	miR-30c-2-3p ↓ miR-326-5p ↓ (from Sema3A inhibitor-treated ischemic As-Exos)	Increasing prostaglandin D_2_ synthase	Suppress astrocyte activation; Promote axonal outgrowth and functional recovery	Hira et al. [65]
	synapsin 1	-	Increase neurite outgrowth and survival; Modulate neuron–glia interaction	Wang et al. [66]
	miR-92b-3p	-	Ameliorate ischemia-induced neuronal apoptosis and injury	Xu et al. [70]
Microglia-derived exosomes	miR-124 from Mi2-Exos)	Targeting USP14	Attenuate ischemic brain injury, neural deficits, apoptosis; Promote neuronal survival	Song et al. [73]
		Downregulating STAT3	Reduce glial scar formation; Improve post-stroke recovery; Inhibit the migration and proliferation of astrocytes	Li et al. [74]
		Increasing Sox2, decreasing Notch1 expression	Promote astrocyte-to-neural progenitor cell transition	
	miRNA-26a (from IL-4 polarized Mi-Exos)	-	Promote angiogenesis	Tian et al. [75]
	miR-424-5p	Regulating the FGF2/STAT3 pathway	Induce cell damage and permeability of BMECs	Xie et al. [76]
Endothelial cell-derived exosomes	miR-126-3p	-	Increase neurite outgrowth; Protect PC12 cells from nerve damage and apoptosis	Gao et al. [81]
	miR-27a miR-19a miR-298 miR-195	Targeting Sema6A, PTEN, and RhoA	Promote the axonal growth of cortical neurons	Zhang et al. [82]
	miR-126	-	Promote axon, myelin, and vascular density and M2 macrophage polarization in diabetic stroke; Improve functional and cognitive functional outcomes	Venkat et al. [85]
Endothelial progenitor cell-derived exosomes	miR-210	Improving mitochondrial function	Improve angiogenic function; Decrease apoptosis and reactive oxygen species production	Ma et al. [84]
	miR-126	Downregulating cleaved caspase-3; Upregulating VEGFR2	Promote neurogenesis and angiogenesis; Improve neurological function recovery	Wang et al. [86]

Abbreviations: CXCL1, chemokine CXC motif ligand 1; PAFR, platelet activating factor receptor; VE-cadherin, vascular endothelial cadherin; eef2k, eukaryotic elongation factor 2 kinase; SOD1, superoxide dismutase 1; ERK1 and ERK2, extracellular signal-regulated protein kinases 1 and 2; Atg7, autophagy-related gene 7; SIRT1, sirtuin 1; AMPK, AMP-activated protein kinase; mTOR, mammalian target of rapamycin; CTSB, cathepsin B; NF-κB, nuclear factor-kappa B; MAPK, mitogen-activated protein kinase; TLR7, toll-like receptor 7; Sema3A, semaphorin 3A; As-Exos, astrocytes-derived exosomes; Mi2-Exos, M2 microglia-derived exosomes; USP14, ubiquitin-specific protease 14; STAT3, signal transducer and activator of transcription 3; IL-4, interleukin-4; FGF2, basic fibroblast growth factor; BMEC, brain microvascular endothelial cell; Sema 6A, semaphorin 6A; PTEN, phosphatase and tensin homolog; RhoA, ras homolog family member A; VEGFR2, vascular endothelial growth factor receptor 2.

**Table 2:** Therapeutic potential of mesenchymal stem cell-derived exosomes.

MSC-Exos	Exosome Cargo	Target	Effects of Dysregulation	References
Bone marrow mesenchymal stem cell-derived exosomes	miR-133b	Downregulating CTGF and RhoA	Improve functional recovery and neurovascular plasticity; Thin the glial scar; Promote neurite outgrowth	Xin et al. [96,97,98]
		Downregulating RABEPK	Secondary release of As-Exos; Enhance post-stroke neurological recovery, neurite outgrowth, and plasticity	Xin et al. [67]
	miR-17-92 cluster	Activating the PI3K/Akt/mTOR/GSK-3β pathway by targeting PTEN	Improve functional recovery and neural plasticity	Xin et al. [99]
		Activating the PTEN/mTOR pathway	Enhance axonal growth	Zhang et al. [100]
	miR-233-3p	Targeting CysLT_2_R	Inhibit M1 microglial polarization-mediated pro-inflammatory response; Improve neurological deficits; Ameliorate ischemic brain injury	Zhao et al. [102]
	miR-138-5p	Downregulating LPCN2	Promote proliferation and inhibit apoptosis of astrocytes; Reduce neurological impairment	Deng et al. [104]
	miR-134	Downregulating the caspase-8-dependent apoptosis pathway	Suppress the apoptosis of oligodendrocytes	Xiao et al. [105]
	miR-132-3p	Repressing RASA1; Activating the Ras/PI3K/Akt/eNOS pathway	Protect endothelial cells from ischemia-induced apoptosis, oxidative stress, and tight junction disruption	Pan et al. [106]
	miR-1906 (from Li-induced MSC preconditioning)	Inhibiting TLR4 and proinflammatory signaling cascades	Enhance neuroregeneration, neuroprotection, and neurological recovery	Haupt et al. [107]
Adipose-derived stem cell-derived exosomes	miR-30d-5p	-	Promote M2 microglia/macrophage polarization; Suppress autophagy	Jiang et al. [113]
	miR-126	Increase doublecortin and FvW levels	Improve neurogenesis, angiogenesis, and functional recovery after stroke; Suppress microglial activation; Inhibit neuroinflammation	Geng et al. [114]
	miR-181-5p	Targeting TRPM7; Upregulating HIF-1α and VEGF; Downregulating TIMP3	Promote the angiogenesis of brain microvascular endothelial cells	Yang et al. [115]
	miR-21-3p (suppressing)	Upregulating MAT2B	Suppress apoptosis and inflammation in neurons	Li et al. [116]
	miR-22-3p	Inhibiting KDM6B-mediated BMP2/BMF axis	Attenuate apoptosis and ischemic brain injury	Zhang et al. [119]

Abbreviations: MSC-Exos, mesenchymal stem cells-derived exosomes; CTGF, connective tissue growth factor; RhoA, Ras homolog family member A; RABEPK, Rab9 effector protein with Kelch motifs; As-Exos, astrocytes-derived exosomes; PI3K, phosphatidylinositol-3-kinase; mTOR, mammalian target of rapamycin; GSK-3β, glycogen synthase kinase 3 beta; PTEN, phosphatase and tensin homolog; CysLT_2_R, cysteinyl leukotriene receptor 2; LPCN2, lipocalin 2; RASA1, protein p120 Ras GTPase-activating protein; eNOS, endothelial nitric oxide synthase; Li, lithium; MSC, mesenchymal stem cell; TLR4, toll-like receptor 4; FvW, von Willebrand factor; TRPM7, transient receptor potential melastatin 7; HIF-1 α, hypoxia-inducible factor 1α; VEGF, Vascular endothelial growth factor; TIMP3, tissue inhibitor of metalloproteinase 3; MAT2B, methionine adenosyltransferase 2B; KDM6B, lysine demethylase 6B; BMP2, bone morphogenic protein 2; BMF, Bcl-2 modifying factor.

## Data Availability

Not applicable.
